# Draft Genome Sequences of Two *Cadophora* Strains Isolated from Water and a Nonalcoholic Beverage Ingredient

**DOI:** 10.1128/MRA.00368-21

**Published:** 2021-07-01

**Authors:** Ewelina Stefanovic, Colm Scully, Jimmy Roader, Finbarr Murphy, Jos Houbraken, Duong Vu

**Affiliations:** a Analytical Services Laboratory, European Refreshments, Ballina, County Mayo, Ireland; b Westerdijk Fungal Biodiversity Institute, Utrecht, the Netherlands; c R&D Operation Brussels, S.A. Coca-Cola Services N.V., Brussels, Belgium; University of California, Riverside

## Abstract

Members of the fungal genus *Cadophora* are isolated from a variety of habitats, including plants, soil, water, food, and indoor environments. Here, we report the draft genome sequences of two strains, Cadophora malorum M34 and *Cadophora* sp. strain M221.

## ANNOUNCEMENT

The genus *Cadophora* was introduced by Lageberg in 1927 with Cadophora fastigiata as the type species (1). Based on phialide morphology similarity, the genus was synonymized with *Phialophora* (2). The initial proposal to reclassify *Phialophora*-like anamorphs according to morphological characteristics (3) was confirmed by the phylogenetic analysis of internal transcribed spacer (ITS) and 28S ribosomal DNA (rDNA) sequences and showed that *Cadophora* represents a separate genus in *Ploettnerulaceae* (*Helotiales*, *Leotiomycetes*) (4). Currently, *Cadophora* encompasses over 20 species (5). *Cadophora* species belong to dark septate endophytes, a polyphyletic group of fungi that produce melanized, septate hyphae (6) associated with plants, are present in soil (6–8), on decaying wood (7, 8), and in water (9), and can survive in extreme habitats, such as Antarctica (10). The two *Cadophora* strains analyzed in this study were isolated from water (Cadophora malorum M34 [=CBS 147375]) and a low-pH ingredient used in a nonalcoholic beverage (*Cadophora* sp. strain M221 [=CBS 147358]) and were deposited in the CBS culture collection at the Westerdijk Fungal Biodiversity Institute, the Netherlands.

Both strains were grown on acidified malt extract agar (5 days at 25°C for M34 and 7 days at 20°C for M221). DNA was isolated with a DNeasy UltraClean microbial kit (Qiagen GmbH, Germany), and genomic libraries were prepared with the Nextera XT DNA library preparation kit (Illumina, Inc., USA). The 250-bp paired-end read sequencing was performed on an Illumina HiSeq platform (MicrobesNG, UK). The *de novo* assembly was performed on samples using SPAdes v3.7 (11), and the quality of the genome assemblies was assessed by QUAST (26). Funannotate v1.7.4 (12) was used for gene prediction and annotation with the protein evidence from a publicly available genome of a *Cadophora* species (13). Specifically, the protein evidence was aligned to the genome using DIAMOND v0.9.24 (14) and Exonerate v2.20 (15). The gene predictions were performed using GeneMark-ES (16), BUSCO 2 (17), AUGUSTUS v3.3.2 (18), and EVidenceModeler (19). The gene models were functionally annotated with Pfam (20), a DIAMOND search on UniProt v2020_24, InterProScan 5 (21), eggNOG v5.0 (22), MEROPS (23), CAZyme (24), BUSCO 2 (dikarya_odb9), and SignalP v5.0 (25).

Sequence assemblies indicated coverages of 203× and 57× for the strains M34 and M221, respectively. The length of the *Cadophora malorum* M34 genome was 48,945,047 bp, consisting of 945 nonoverlapping contigs, with a contig *N*_50_ value of 121,946 bp and a maximum contig size of 931,894 bp. A total of 16,400 protein-coding genes were identified, along with 107 RNAs. In the case of *Cadophora* sp. M221, the assembly yielded a genome sequence of 60,032,319 bp, consisting of 6,664 contigs. The maximum contig size was 316,907 bp, and the contig *N*_50_ value was 47,756 bp, while a total of 17,654 protein-coding genes and 124 tRNAs were identified. The GC content of the genomes was 48.01% for M34 and 46.46% for M221. A summary of genome completeness using BUSCO 2 (dikarya_odb9) revealed 98.6% complete, 98.2% complete and single-copy, 0.4% complete and duplicated, 0.8% fragmented, and 0.6% missing orthologs for M34. For M221, these values were 98.1%, 97.7%, 0.4%, 1.4%, and 0.5%, respectively.

These sequencing data present a valuable addition to the set of currently available *Cadophora* genomes and enable deeper understanding of the features and further comparative genome analysis of strains of this genus.

### Data availability.

The whole-genome shotgun projects have been deposited at DDBJ/EMBL/GenBank under the accession numbers JAFJYH000000000 for M34 and JAFJYG000000000 for M221. The raw reads have been deposited under the SRA accession numbers SRR14354866 and SRR14354867 for M34 and SRR14354808 and SRR14354809 for M221.
